# Molecular characterization of *Neisseria meningitidis* isolates recovered from patients with invasive meningococcal disease in Colombia from 2013 to 2016

**DOI:** 10.1371/journal.pone.0234475

**Published:** 2020-07-14

**Authors:** Jaime Moreno, Zonia Alarcon, Eliana Parra, Carolina Duarte, Olga Sanabria, Diego Prada, Jean Marc Gabastou

**Affiliations:** 1 Grupo de Microbiología, Instituto Nacional de Salud (INS), Bogotá, Colombia; 2 Panamerican Health Organization/World Health Organization (PAHO/WHO), Washington, DC, United States of America; Defense Threat Reduction Agency, UNITED STATES

## Abstract

**Background:**

*Neisseria meningitidis* is a significant cause of morbidity and mortality worldwide. Meningococcal isolates have a highly dynamic population structure and can be phenotypically and genetically differentiated into serogroups and clonal complexes. The aim of this study was to describe the phenotypic and genotypic characteristics of invasive isolates recovered in Colombia from 2013 to 2016.

**Methodology:**

A total of 193 invasive isolates were analyzed. Phenotypic and genotypic characteristics were determined by serotyping, antimicrobial susceptibility testing, pulsed-field gel electrophoresis (PFGE) and whole-genome sequencing.

**Results:**

Based on the results, meningococcal serogroups C, B and Y were responsible for 47.9%, 41.7%, and 9.4% of cases, respectively, and the distribution of serogroups B and C changed over time. Fifteen clonal groups and 14 clonal complexes (cc) were identified by PFGE and genome sequencing. The main clonal group included serogroup B isolates with sequence type (ST)-9493 and its four single-locus variants, which has only been identified in Colombian isolates. The clonal population structure demonstrates that the isolates in this study mainly belong to four clonal complexes: ST-11 cc, ST-32 cc, ST-35 cc and ST-41/44 cc. Thirty-eight *pen*A alleles were identified, but no correlation between MICs and specific sequences was observed.

**Conclusion:**

This study shows that most meningococcal isolates recovered from patients with invasive meningococcal disease in Colombia are strains associated with distinct globally disseminated hyperinvasive clones.

## Introduction

*Neisseria meningitidis* is a cause of invasive meningococcal disease (IMD) that is associated with outbreaks of epidemic and endemic infections, with high morbidity and mortality worldwide [1]. Although the natural reservoir of *N*. *meningitidis* is the human upper respiratory tract, it can invade the bloodstream and is the cause of meningitis in 30%-60% of cases; it also causes meningococcemia at a frequency of 20%–30%, which is fatal in 50–80% of untreated cases [2,3]. There are approximately 1.2 million cases of meningococcal infection each year, with 335,000 deaths occurring worldwide [1]. The annual incidence of meningococcal disease in Latin America varies widely, ranging from <0.1 cases per 100,000 inhabitants in countries such as Bolivia, Cuba, Mexico, Paraguay, and Peru to nearly two cases per 100,000 inhabitants in Brazil [4]. In Colombia, the estimated annual incidence in 2018 was 0.6 cases per 100,000 inhabitants and 0.7 cases per 100,000 children less than 5 years old [5].

Isolates of *N*. *meningitidis* are classified into twelve serogroups based on capsular polysaccharide composition, but only six serogroups (A, B, C, W, X, and Y) are associated with IMD [3]. Nonetheless, the proportion of serogroups varies notably across countries and age groups. Globally, serogroup B has been the most prevalent, whereas serogroup X isolates have been the least frequently reported cause of IMD [6]. *N*. *meningitidis* has a highly dynamic population structure due to horizontal gene transfer, and genotypic characterization has allowed for an understanding of the epidemiology and population biology of meningococcus [1]. The relationships among isolates of evolving microbial populations can be revealed by molecular characterization methods such as MLST, which groups related isolates into clonal complexes (ccs) [7]. However, a limited number of ccs are responsible for the majority of meningococcal infections globally and are termed hyperinvasive ccs, of which the most frequent are those belonging to the sequence type (ST) -5 cc (Lineage 10), ST-32 cc (Lineage 5), ST-41/44 cc (Lineage 3), and ST11 cc (Lineage 11) [8].

In Colombia, passive and voluntary surveillance of *N*. *meningitidis* is performed by the National Health Institute (NHI) as part of the Network Surveillance System for the Causative Agents of Pneumonia and Meningitis (SIREVA II) program of the Pan American Health Organization (PAHO) [9]. This surveillance program receives isolates from Public Health Laboratories around the country. Surveillance activities include characterizing isolates in terms of antimicrobial susceptibility, serogroup, and subtyping, as well as some special studies, such as research on the increase in the circulation of serogroup Y isolates [10], outbreak investigation on IMD [11] and characterization of carriage isolates [12]. Between 1987 and 2018, 985 isolates were submitted to the NHI, of which serogroup B was confirmed in 595 (60.4%), serogroup C in 252 (25.6%), serogroup Y in 107 (10.9%), serogroup W in 7 (0.7%), serogroup X in 3 (0.3%) and 21 (2.1%) were nongroupable. However, since 2016, serogroup C isolates have increased in frequency: of the 171 isolates submitted from 2016 to 2018, serogroup C accounted for 67.2% (n = 115) [5]. Although universal meningococcal vaccination has not been introduced in Colombia as part of national immunization programs; however, tetravalent conjugate vaccines of serogroups A, C, Y, and W are available. Additionally, studies related to the clonal diversity of *N*. *meningitidis* associated with invasive disease have not been performed. Therefore, the aim of this study was to characterize the population structure of invasive *N*. *meningitidis* isolates recovered from patients with IMD between 2013 and 2016.

## Materials and methods

### Isolates

A total of 193 *N*. *meningitidis* isolates were recovered from 2013 to 2016 through the national network of Public Health Laboratories as part of laboratory-based passive and voluntary surveillance for acute bacterial meningitis and acute respiratory infection performed by the Microbiology Group of the NHI, which is part of the SIREVA II [9]. This study did not include culture-negative or PCR-diagnosed IMD cases. The isolates were identified at local laboratories and transported in Amies transport medium to the Microbiology Group for confirmation, serogrouping, and antimicrobial susceptibility testing. Isolates were plated on tryptic soy medium supplemented with 5% sheep blood and incubated at 37 °C in 5% CO_2_ for 24 hours and identified by standard methods of colony morphology, gram staining, oxidase testing and biochemical profiling. The serogroup of the isolates was determined by the slide agglutination assay using commercial antisera (DIFCO, Becton Dickinson) [13]. Antimicrobial susceptibility testing for penicillin, ceftriaxone, ciprofloxacin, chloramphenicol, and rifampicin was performed by microdilution testing on agar and by concentration gradient strips (E-test-BioMérieux) according to Clinical and Laboratory Standards Institute guidelines [14].

### Pulsed-field gel electrophoresis (PFGE)

Genotype identification was performed by pulsed-field gel electrophoresis (PFGE) using the *SpeI* restriction enzyme, as based on a previously published protocol [13]. The reference strain *N*. *meningitidis* serogroup B, ATCC 13090, was used as a control. The genetic relationship among the PFGE patterns was generated by the program Gel Compare II (Bio-Rad), and a dendrogram of PFGE patterns was constructed using the unweighted pair group with the arithmetic averaging (UPGMA) method and the Dice similarity coefficient. A cluster in the dendrogram was considered at ≥ 80% genetic relationship. Clonal groups were designated with capital letters.

### Whole-genome sequencing

Complete genome sequencing was performed on 110 isolates, which were selected according to the clusters generated by PFGE, antimicrobial susceptibility profiling, and association with meningococcal outbreaks. The selection included serogroup B (n = 75), C (n = 28), Y (n = 5), W (n = 1) and nongroupable (n = 1) isolates. Genomic DNA extraction was performed using QIAamp DNA Mini Kit (QIAGEN) following the manufacturer’s instructions. Genomic DNA was sequenced using an Illumina MiSeq sequencer. Short-read sequences were assembled *de novo* using SPAdes v3.1 [15]. The draft genome was annotated using Prokka v1.4.0, an open-source software tool [16]. Pangenome analysis was performed with Roary v1.0 [17]. The results were visualized using Phandango (http://jameshadfield.github.io/phandango/), and trees were visualized using the FigTree software program (http://tree.bio.ed.ac.uk/software/figtree/). Multilocus sequence typing (MLST) was carried out via extraction of whole-genome sequencing data using the software MLST v1.8 and analyzed with the Neisseria PubMLST database (http://pubmlst.org/neisseria/) [7]. MLST is a widely used molecular typing method, which recognizes individual meningococcal isolates as a ST and groups of genetically related STs as cc [18]. Novel sequence types were submitted to the PubMLST database for curation. MLST data were analyzed using the eBURST program. eBURST divides an MLST data set into groups of related isolates and cc. Genotypes that differ at only one MLST loci are called single-locus variants (SLVs) and genotypes that differ at two loci are termed double-locus variants (DLVs) [19]. The *pen*A gene, which encodes penicillin-binding protein 2, was examined.

### Statistical analysis

A descriptive analysis was performed. Data were analyzed using Microsoft Excel and Epi Info 7 software. The relationship or association between qualitative variables was evaluated using the Chi-square test or Fisher’s exact test. A p-value ≤ 0.05 was considered to indicate a significant association.

## Results

During the 4-year study period, 17 of the 33 Public Health Laboratories submitted 193 isolates from cases of laboratory-confirmed IMD, with Bogotá (31.1%), Antioquia (20.2%), Bolívar (14.5%) and Valle (9.8%) provided 75.6% of the isolates. However, the isolates do not represent all the cases that occurred during the study period. The proportion of *N*. *meningitidis* isolates increased from 18.6% in 2013 to 34.7% in 2016 (p = 0.0002). The main diagnoses reported were meningitis (66.8%) and meningococcemia (26.4%). The patients' ages ranged from 17 days to 89 years, and isolates were recovered more frequently from patients aged 20 to 49 (26.9%) and 6–19 (23.8%) years (Table 1).

**Table 1:** Distribution of the PFGE patterns, sequence types (ST), serogroup, year and age of the isolates received in the surveillance program in Colombia from 2013 to 2016.

PFGE pattern	n	Serogrup					Year				Age						Diagnosis				Susceptibility to penicillin			MLST
		B	C	Y	W	NST	2013	2014	2015	2016	<1	1–5	6–19	20–49	>50	ND	Meningitis	Meningo-coccemia	Other	ND	S	I	R	
A	36		36				4	5	2	25	4	2	7	17	5	1	21	12	1	2	22	14		11, 11149, 14186, 14189
B	27	27					9	11	7		2	9	6	6	4		20	5		2	19	8		9493, 14185, 14188, 14190
C	26		26						17	9	1	5	6	7	4	3	15	10		1	19	7		11, 14192
D	10		3	7			1		4	5	1	2	2	1	3	1	5	3	2		8	2		11, 23, 14193
E	7	7					5	1	1		3		3			1	5	2			2	5		35, 3992,
F	6		6						3	3		1	4	1			6				1	5		2561
G	5		5					1	2	2	1	2	1	1			4	1			0	5		14184
H	4		4					1	3		1	1	2				2	2			0	4		278
I	4	1	1	2				2		2		1	2			1	3		1		1	3		23, 13977
J	3		3				2			1		1		1	1		3				1	2		178
K	3	3								3				3			2			1	2	1		1383, 14194
L	3			3				3						1	2		2	1			2	1		5024
M	3	3					1	2				1		2			3				3			5682, 9493
N	3	3					1	2			2					1	3					3		32, 33
O	3	2	1				1		1	1	2	1					2	1			2	1		11, 33, 35
NR	50	34	9	5	1	1	12	14	8	16	13	3	13	12	8	1	33	14	1	2	20	29	1	*24 STs
**Total**	193	80	94	17	1	1	36	42	48	67	30	29	46	52	27	9	129	51	5	8	102	90	1	43 ST´s
	%	41.4	48.7	8.8	0.5	0.5	18.6	21.7	24.8	34.7	15.5	15.0	23.8	26.9	13.9	4,6	66.8	26.4	2.5	4.3	52.8	46.6	0.6	

ND: No data; NR: Not related.

* STs. 32, 33, 53, 84, 213, 409, 485, 1383, 1434, 1624, 2288, 2561, 3128, 4237, 9193, 9461, 9493, 11311, 13974, 13975, 13976, 14183, 14187 and 14191.

**Fig 1:**
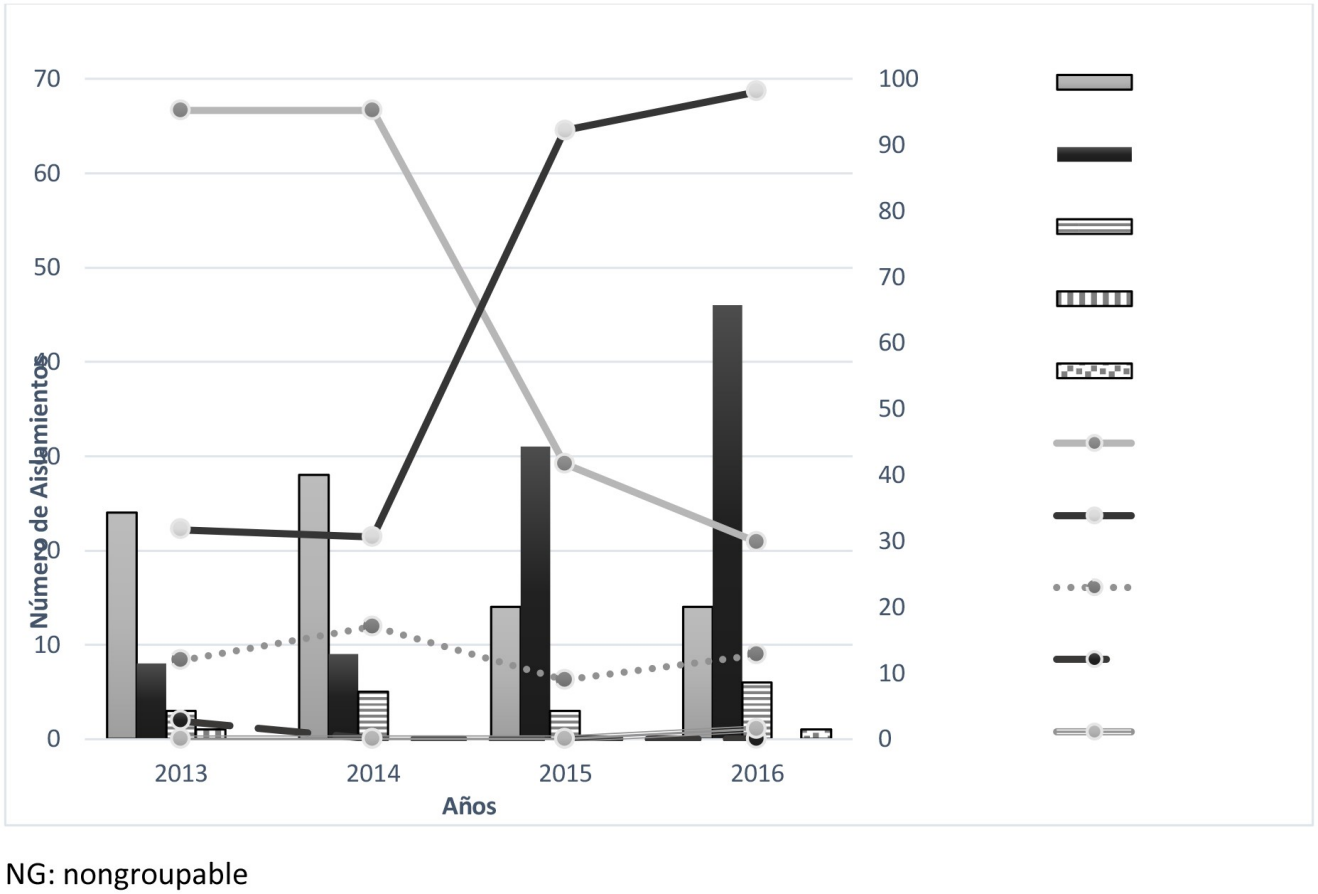
Serogroup distribution of meningococcal disease in Colombia from 2013 to 2016.

The most common serogroup found was serogroup C (n = 94; 48.7%), and the proportion of *N*. *meningitidis* C (MenC) isolates increased from 22.2% in 2013 to 68.6% in 2016 (p < 0.002). *N*. *meningitidis* B (MenB) was the second most predominant serogroup (n = 80; 41.4%), exhibiting a reduction in frequency from 66.7% in 2013 to 20.9% in 2016 (p < 0.002). Serogroups Y (n = 17; 8.9%) and W (n = 1; 0.5%) were less frequently found. Only one isolate was nongroupable (0.5%) (Fig 1).

### Molecular typing

PFGE was performed on all isolates, and analysis of the restriction patterns allowed the differentiation of 15 clonal clusters that grouped 143 (74.1%) isolates (Table 1). Whole-genome sequencing was performed for 110 isolates, and MLST analysis identified 43 STs, 16 of which had not been previously identified (Table 2) (S1 Fig).

**Table 2:** Distribution of clonal complexes, sequence types (ST) and serogroups of the isolates received in the surveillance program in Colombia from 2013–2016.

Clonal Complex	ST	n	Serogroup					Total
			B	C	Y	W	NST	
ST-9493 Complex	9493	27	27					34
	13974	1	1					
	13976	1	1					
	13977	1	1					
	14185	1	1					
	14188	1	1					
	14190	1	1					
	14191	1	1					
ST-11 complex/ET-37 complex	11	16		16				20
	11149	1		1				
	14186	1		1				
	14189	1		1				
	14192	1		1				
ST-32 complex/ ET-5 complex	32	3	3					10
	33	6	6					
	5682	1	1					
ST-35 complex	35	7	7					9
	278	1		1				
	3992	1	1					
ST-41/44 complex/Lineage 3	409	1	1					9
	485	3	3					
	2288	4	4					
	14187	1	1					
ST-269 complex	2561	3	1	2				5
	9461	2	2					
ST-178 complex	178	1		1				4
	3128	2		2				
	14183	1	1					
ST-23 complex/ Cluster A3	23	2			2			4
	5024	1			1			
	14193	1			1			
ST-60 complex	1383	3	3					4
	14194	1	1					
ST-213 complex	213	1	1					2
	9193	1	1					
ST-167 complex	1624	1			1			1
ST-22 complex	184	1				1		1
ST-4821 complex	11311	1	1					1
ST-53 complex	53	1					1	1
ST-865 complex	4237	1	1					1
No determinated	1434	1	1					4
	13975	1	1					
	14184	2		2				

Electrophoretic pattern A grouped 36 (18.7%) serogroup C isolates (similarity of 81.3%). This cluster included isolates belonging to ST-11, ST-11149, ST-14186, and ST-14189 of cc11. Intermediate sensitivity to penicillin was observed for 14 (29.7%) isolates. This clonal group was more frequently recovered in 2016 (n = 25, 69.4%) and in patients aged from 20 to 49 years (n = 17, 47.2%). Twenty-seven serogroup B (13.9%) isolates (MenB) with similar PFGE patterns were grouped into pattern B (similarity of 80%). These were associated with ST-9493 and seven new STs: four single-locus variants (SLVs) ST-13974, ST-14185, ST-14188 and ST-14190; and three double-locus variants (DLVs) ST-13976, ST-14191, and ST-13977. The majority of isolates (n = 17; 59.2%) were collected from patients in the region of Bolívar, including patients of an IMD outbreak [10]. Cluster C included 26 (13.5%) serogroup C isolates (MenC) and ST-11 recovered during 2015 and 2016. Pattern D was associated with 10 (5.2%) isolates: seven of serogroup Y and three of MenC related to ST-23 and ST-11, respectively. Electrophoretic pattern E grouped seven (3.6%) MenB isolates belonging to ST-35 and ST-3992 of cc35. Pattern F involved six (3.1%) MenC isolates associated with ST-2561 of the ST-269 complex. Pattern G was associated with five (2.6%) MenC isolates, intermediate sensitivity to penicillin, and new ST-14184 and DLV ST-13975. Patterns H (2.1%) and J (1.6%) were formed by isolates with MenC isolates and were genetically associated with ST-35 and ST-178, respectively. Seven other minor clonal groups were identified (I, K–O), grouping 19 (9.8%) isolates associated with different STs. The remaining 50 (24.9%) isolates were not clonally related (NR) based on PFGE; 12 different STs were identified in 33 (17.1%) isolates, including the new ST-13975 in one serogroup B isolate.

**Fig 2:**
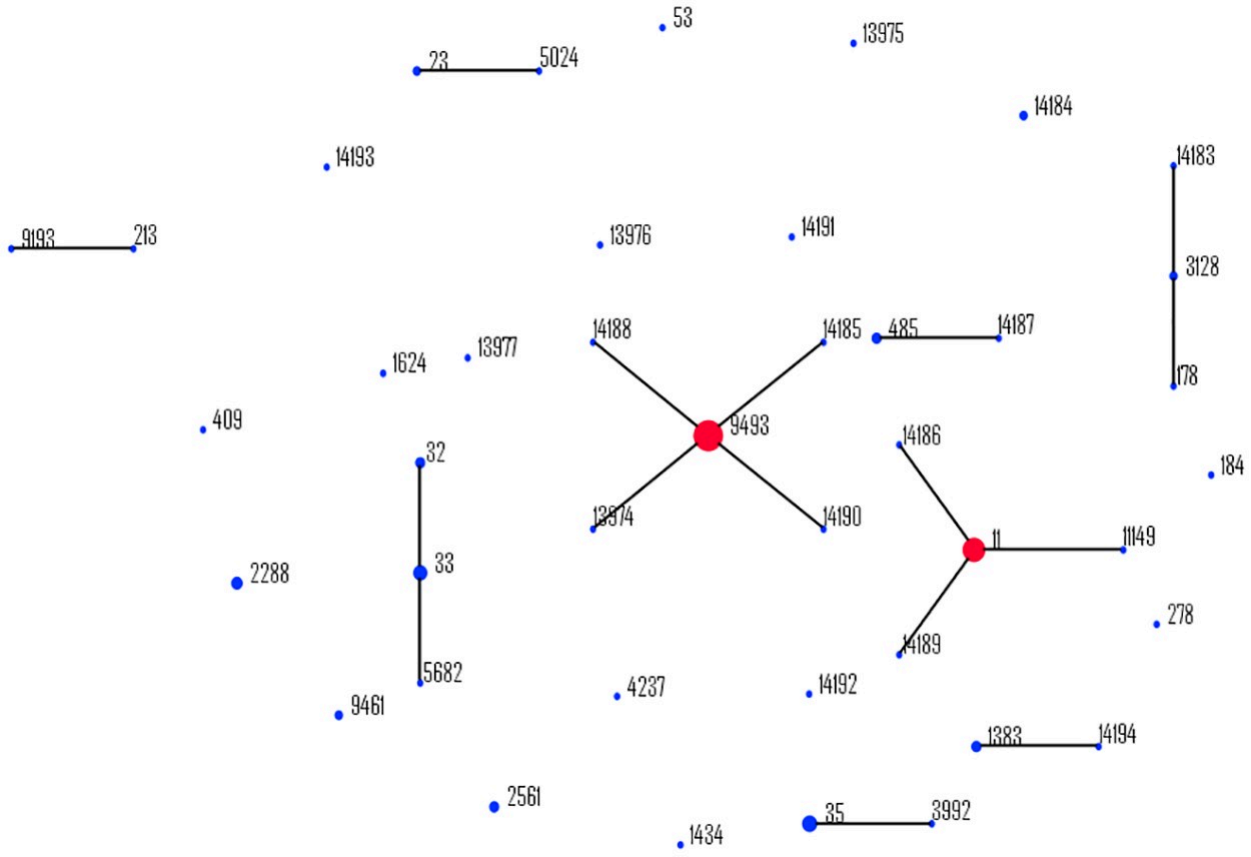
Distribution of sequence types (STs) among the clonal complexes of *N*. *meningitidis*. Genetic relatedness was determined by full eBURST analysis using MLST allelic profiles of seven housekeeping genes. STs that are linked by a line belong to the same cluster. Circle sizes are proportional to the number of isolates within the ST.

Among 110 isolates, 43 STs were found according to MLST analysis, 16 of which were novel (ST-13974, ST-13975, ST-13976, ST-13977, and ST-14183 to ST-14194) (Table 2). eBURST analysis using the group definition of only isolates that share identical alleles at six or seven MLST loci grouped these STs into 9 ccs and 18 singletons (Fig 2). In addition, eBURST analysis of the isolates with group definitions of 5 or more matches revealed 12 ccs and 7 singletons. These results indicate great genetic diversity among the isolates. ST-9493 was the main clonal group, with 34 (31.0%) serogroup B isolates, and eBURST analysis using data from the *Neisseria* PubMLST database showed that ST-9493 is an SLV of ST-136, differing from ST-41 at three loci. The remaining isolates studied were associated with 14 international ccs, of which the most frequent was the ST-11 cc (n = 20; 18.2%), followed by the ST-32 cc (n = 10; 9.1%), ST-35 cc (n = 9; 8.2%) and ST-41/44 cc (n = 9; 8.2%). Four isolates belong to a cc not currently assigned (Table 2). In general, there was an association of some ccs with a particular serogroup, with some exceptions: the ST-35 cc was found to predominantly be serogroup B (n = 8; 89%) with ST-35 and ST-3992, and the remaining isolate belongs to serogroup C ST-278. The ST-269 cc comprised ST-2561 with MenB (n = 1) and MenC (n = 2) isolates and ST-9461 with two MenB isolates. The ST-178 cc was formed by isolates with serogroup C ST-178 and ST-312 and serogroup B with new ST-14183.

**Fig 3:**
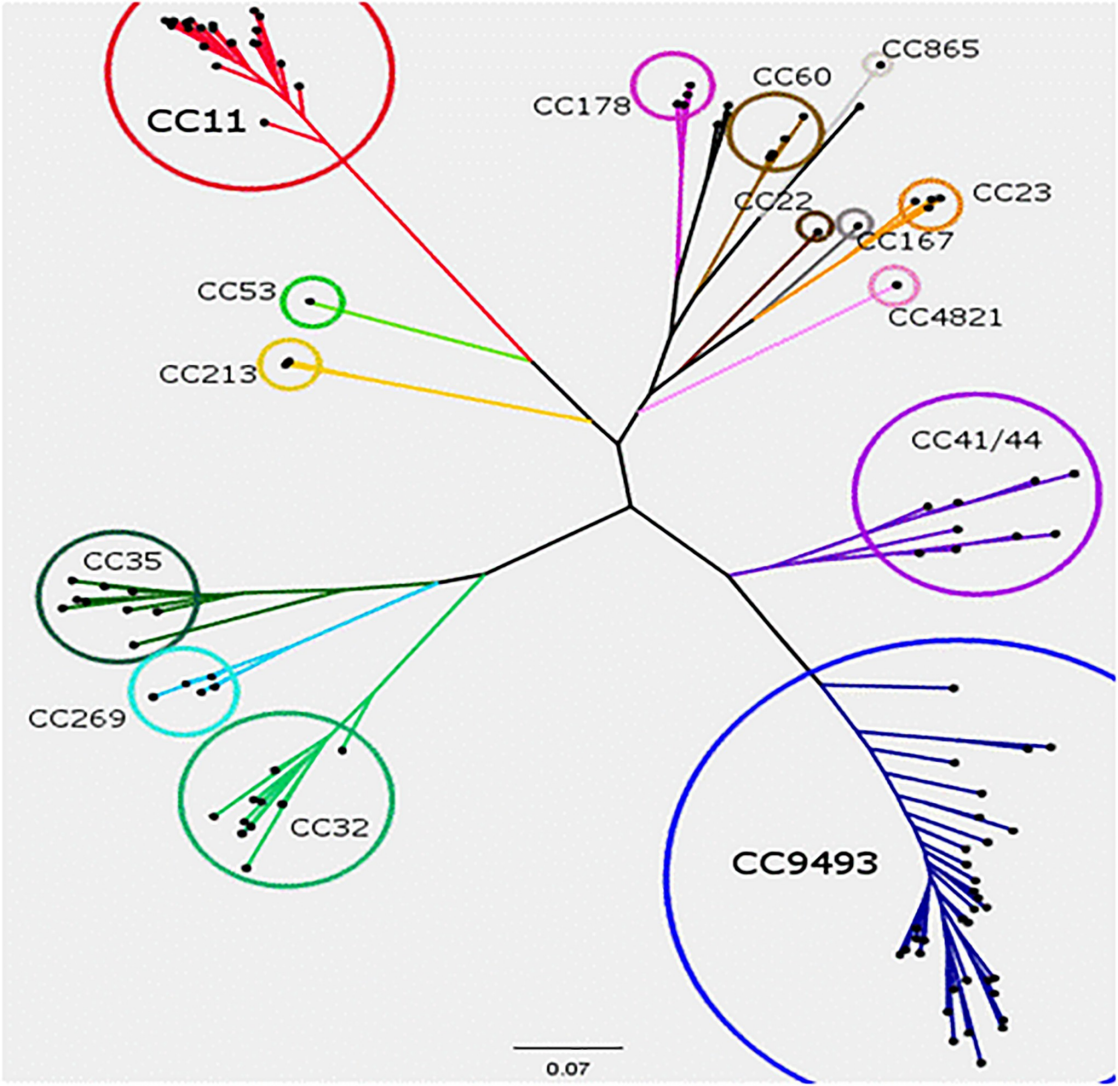
Relationships between the major clonal complexes (ccs) of *N*. *meningitidis* isolates recovered from cases of invasive disease in Colombia from 2013 to 2016. A maximum likelihood tree was constructed using the whole-genome sequences of 110 *N*. *meningitidis* isolates analyzed in this study. Branch lengths represent the genetic distance, and ccs are shown in colors.

Genetic relatedness between isolates was assessed by aligning the core genes present in all isolates and generating a maximum likelihood phylogeny (Fig 3). The phylogenetic network generated indicated high genetic diversity, with a clonal population structure mainly composed of four clonal complexes: ST-11 cc, ST-32 cc, ST-35 cc and ST-41/44 cc. ST-9493 isolates were grouped in a subcluster and placed in a branch of the tree separated from the ST-41/44 cc, indicating a possible new clonal group related to serogroup B. Genomic sequences have been deposited in NCBI under the project accession number PRJNA407579.

### Antimicrobial susceptibility

All isolates were found to be susceptible to ceftriaxone, ciprofloxacin, chloramphenicol, and rifampicin. However, 43.0% (n = 83) of the isolates of all serogroups from 2014 presented intermediate sensitivity to penicillin (Minimum inhibitory concentration (MIC) range 0.125–0.25 μg/mL), whereas one isolate of serogroup Y recovered in 2016 was resistant (MIC of 0.5 μg/mL). Analyses of *pen*A sequences revealed the presence of 38 alleles among all isolates sequenced. The most frequent alleles were as follows: *pen*A2189 (n = 22; 20%), with MIC values ranging from 0.03 to 0.125 μg/mL, identified in ST-9493 isolates; *pen*A25 (MIC range 0.03 to 0.125 μg/mL), observed in 18 (16.4%) ST-11 cc isolates; and *pen*A61 (n = 9; 8.1%) in isolates of different ccs (ST-60 cc, ST-22 cc, ST-269 cc) and exhibiting MIC values between 0.03 to 0.125 μg/mL. Only one serogroup Y ST167 isolate with a MIC of 0.5 μg/mL carried *pen*A2208. No obvious correlation between individual MICs and any specific *pen*A sequence was found.

## Discussion

In this study, phenotypic and genotypic characterization of the population structure of invasive *N*. *meningitidis* in Colombia from 2013 to 2016 was carried out using isolates recovered from the national surveillance program around the country. The most common serogroup was serogroup C, which was associated with the ST-11 cc, followed by serogroup B related to the ST-9493 cc, as well as the ST-32 cc and ST-41/44 cc. Phylogenetic analysis demonstrated the circulation of some clonal complexes and isolates with ST-9493 being the predominant lineage.

Meningitis is the most common form of IMD, accounting for 30%–60% of all cases, whereas septicemia is the predominant presentation in 20–30% of cases [20]. In this study, isolates were recovered from laboratory-based surveillance that included only the reporting of meningitis cases, but some cases of meningococcemia were incorporated in this surveillance period. Since 2016, other meningococcal diagnoses have been included in mandatory surveillance, and the cases should be reported to improve our knowledge regarding the clinical presentation of these infections and to enable a better public health response [21].

There are geographical differences in the distribution of meningococcal serogroups [3]. Serogroup C was the most common serogroup identified during the reporting period, followed by serogroup B, which was prevalent during previous periods of surveillance. In Latin America, MenB and MenC isolates are responsible for the majority of cases reported in the region, with an increased number of MenW isolates being observed in Chile and Argentina [22]. MenB isolates were reportedly the predominant cause of IMD in the USA, Canada, and nearly all countries in Europe, and MenY has been the cause of many cases in Nordic countries [3].

Our molecular characterization showed a high level of heterogeneity among invasive meningococcal isolates. In recent years, serogroup C has been the most prevalent serogroup of MD cases in Colombia [23]. In this study, cases of IMD serogroup C were caused by diverse meningococcal strains, representing 9 different PFGE clonal groups associated with four major ccs: ST-11, ST-178, ST-269, and ST-35. The majority of invasive serogroup C isolates were the ST-11 cc, and the expansion of this clone coincides with the observed increasing incidence of meningococcal C disease. Meningococci belonging to the hyperinvasive ST-11 cc are associated with high levels of morbidity and mortality and can cause sporadic disease cases or regional outbreaks, predominantly affects adolescents and young adults and may express serogroups C, W, B or Y [24]. The MenC ST-11 cc has been responsible for sporadic cases or outbreaks in several countries, even after the introduction of the meningococcal serogroup C conjugate vaccine [25,26]. Capsular switching events from C to B or W strains are not rare within the cc11 [24,27]. However, in contrast to findings obtained in other countries [25,28], all isolates associated with the ST-11 cc in this study were serogroup C. ST-178 was previously reported in nongroupable meningococcal carriage isolates collected in students aged 15–21 years in Colombia [12], in military recruits in Finland [29] and in students in the United Kingdom [30], but only one invasive isolate was found in the PubMLST database (http://pubmlst.org/software/database/bigsdb Accessed 16 November 2019). Meningococcal ccs differ in their pathogenic potential: some are associated with disease, whereas others are associated with carriage. Nonetheless, horizontal gene exchange and recombinant events within ccs in the human nasopharynx can result in antigenic diversity, which can result in isolates with invasive potential acquiring capsular genes and may, therefore, express different serogroups [31,32]. The identification of ST-178 isolates with serogroups C and B highlights the importance of surveillance of both carried and disease-causing meningococcal isolates.

Genotypically, the isolates of serogroup B were more diverse than those of serogroup C, being grouped into 7 clonal groups and 34 unique electrophoretic patterns related to 9 ccs. The main clonal group was ST-9493, originally described in one carriage isolate from Colombia and without an assigned cc (http://pubmlst.org/software/database/bigsdb; accessed 16 November 2019). ST-9493 was generated by replacing the *gdh* allele of the original ST-136 (ST-41/44 cc), which differs from ST-41 and ST-44 at three loci and has been found almost exclusively in healthy carriers [33]. ST-9493 was identified in isolates collected from patients in an outbreak of IMD in Colombia, related to ST-41/44 cc [12], and from patients in different regions of the country, demonstrating the local expansion of this ST. Several studies have shown the emergence of new STs of *N*. *meningitidis* serogroup B are associated with outbreaks or endemic disease, and it may be considered as a new meningococcal cc [34–36]. New STs may arise by horizontal gene transfer between circulating *N*. *meningitidis* strains and recombination events [37]. It is possible that clonal group ST-9493 is associated with the ST-41/44 cc or represents a new cc; however, additional genome analysis is required to more accurately reveal relationships among the strains. The second most prevalent cc was ST-32, which was described in northern Norway in 1969 and is currently known to have a wide global distribution [38,39]. Analysis of whole-genome sequence data of a global collection of isolates indicated that ST-32 cc serogroup B *N*. *meningitidis* was not the result of the emergence of a single invasive clone; rather, this serogroup was the result of multiple distinct localized outbreaks [39]. Other dominant ccs of serogroup B were identified in this study. The ST-35 cc accounted for isolates of serogroups B and C, similar to that reported for invasive strains from children in Tunisia [40] or from the strain collection between 2015 and 2017 in the Czech Republic [41]. The ST-41/44 cc associated with serogroup B is currently the most common complex in the PubMLST dataset, and it has considerable genetic diversity and exhibits a rapid evolutionary change in genotype [42]. The ST-269 cc is frequently associated with community outbreaks in Europe [35,42]. Two multicomponent meningococcal B vaccines have been licensed for the control of serogroup B disease; therefore, it is necessary to examine the genetic diversity and distribution of meningococcal vaccine targets to provide baseline data before newer vaccines are introduced into the population.

CC23 and CC167 were associated with serogroup Y, similar to the results of Abad et al. in a study examining the molecular epidemiology of serogroup Y IMD in Latin America [43]. High-resolution genetic analyses have emphasized the high degree of genetic similarity between carriage and invasive serogroup Y isolates and the clonal stability of the ST-23 cc over time [44]. During the study period, only one serogroup W ST-22 cc isolate was recovered, and it is different from the ST-11 cc isolates observed in Europe, China and South American Cone countries, where it has emerged as a leading cause of IMD [24].

Globally, resistance to antibiotics in *N*. *meningitidis* isolates is relatively rare, but the emergence of meningococcal strains with decreasing susceptibility to antibiotics is of increasing public health concern [45]. Alterations in the gene encoding PBP2 (*pen*A) are associated with reduced affinity to penicillin and thus a decrease in susceptibility to the antibiotic [46]. In this study, there was no association between reduced susceptibility to penicillin and the presence of *pen*A alleles. Overall, reduced susceptibility to penicillin may be multifactorial and arise from yet-unidentified mechanisms other than mutations in the *pen*A gene [47]. Future comparison of core and accessory genes is required to elucidate this issue.

The present study has several limitations. First, although all *N*. *meningitidis* cases should be reported, some cases might have been missed because of difficulty in recovering the organism, because the isolates were not sent correctly or because they were not referred to a National Reference Laboratory. Second, the study did not include culture-negative and PCR-diagnosed IMD cases, which represent 28.9% of all cases submitted to NHI between 2015 and 2018 [5]. Third, most of the isolates (66.8%) in this study were recovered from meningitis cases because only the report of this pathology was mandatory in the national surveillance program. However, this study provides the first insight into the molecular characterization of invasive *N*. *meningitidis* isolates in Colombia.

In conclusion, the present study reveals a diverse genetic background among the meningococcal population of invasive isolates recovered in Colombia. We observed increasing amounts of the hyper-invasive MenC cc11, and the emergence of a novel sequence type (ST-9493) which was associated with the non-immunogenic serogroup B. Further work will be required to determine its potential susceptibility to MenB vaccines. Additionally, we observed IMD caused by of a previously commensal sequence type (ST-178) which can express at least two different serogroups. Analysis based on genomic sequencing allows for determining relationships among isolates and monitoring circulating and emerging strains provides a basis for evidence-based decision making on the use of meningococcal vaccines, and improves our understanding of the population structure and evolution of *N*. *meningitidis*.

## Supporting information

S1 Fig Dendrogram of invasive *N*. *meningitidis* isolates from 2013–2016. (DOC)

S1 Table *N*. *meningitis* clonal complex distribution by serogroup for Colombia, 2013–2016. (DOCX)
