# Supplementary material for: Molecular characterization of Neisseria meningitidis isolates recovered from patients with invasive meningococcal disease in Colombia from 2013 to 2016
Source: PLoS One. 2020 Jul 14;15(7):e0234475. doi: 10.1371/journal.pone.0234475 (PMC7360035; doi:10.1371/journal.pone.0234475)
Supplement: S1 Table — (DOCX) [file pone.0234475.s002.docx]

| **Clonal complexes** | **ST** | **n** | **%** | **Serogroup** | | | | | **Year** | | | | |
| --- | --- | --- | --- | --- | --- | --- | --- | --- | --- | --- | --- | --- | --- |
|  |  |  |  | **B** | **C** | **Y** | **W** | **NST** | **2013** | **2014** | **2015** | **2016** | **2017** |
| ST-41/44 complex/Lineage 3 | 9493 | 34 | 29.6 | 34 |  |  |  |  | 9 | 13 | 10 | 1 | 1 |
|  | 485 | 5 | 4.3 | 5 |  |  |  |  | 1 | 2 | 1 |  | 1 |
|  | 2288 | 4 | 3.5 | 4 |  |  |  |  | 1 | 1 |  | 2 |  |
|  | 409 | 1 | 0.9 | 1 |  |  |  |  |  | 1 |  |  |  |
|  | 2851 | 1 | 0.9 | 1 |  |  |  |  |  |  |  | 1 |  |
| ST-11 complex/ET-37 complex | 11 | 18 | 15.7 |  | 17 | 1 |  |  |  |  | 4 | 14 |  |
|  | 11149 | 3 | 2.6 |  | 3 |  |  |  |  | 1 |  | 1 | 1 |
| ST-32 complex/ ET-5 complex | 32 | 3 | 2.6 | 3 |  |  |  |  |  | 2 |  | 1 |  |
|  | 33 | 6 | 5.2 | 6 |  |  |  |  | 3 | 2 | 1 |  |  |
|  | 5682 | 1 | 0.9 | 1 |  |  |  |  |  | 1 |  |  |  |
| ST-35 complex | 35 | 7 | 6.1 | 7 |  |  |  |  | 5 | 1 |  | 1 |  |
|  | 278 | 1 | 0.9 |  | 1 |  |  |  |  |  | 1 |  |  |
|  | 3992 | 1 | 0.9 | 1 |  |  |  |  |  |  | 1 |  |  |
| ST-269 complex | 2561 | 3 | 2.6 | 1 | 2 |  |  |  |  |  |  | 3 |  |
|  | 9461 | 2 | 1.7 | 2 |  |  |  |  |  |  |  | 2 |  |
| ST-23 complex/Cluster A3 | 23 | 3 | 2.6 |  |  | 3 |  |  |  |  |  | 3 |  |
|  | 5024 | 1 | 0.9 |  |  | 1 |  |  |  | 1 |  |  |  |
| ST-178 complex | 178 | 1 | 0.9 |  | 1 |  |  |  | 1 |  |  |  |  |
|  | 3128 | 3 | 2.6 | 1 | 2 |  |  |  | 2 |  |  | 1 |  |
| ST-60 complex | 1383 | 4 | 3.5 | 4 |  |  |  |  | 1 |  |  | 3 |  |
| ST-213 complex | 213 | 1 | 0.9 | 1 |  |  |  |  |  | 1 |  |  |  |
|  | 9193 | 1 | 0.9 | 1 |  |  |  |  | 1 |  |  |  |  |
| ST-22 complex | 184 | 1 | 0.9 |  |  |  | 1 |  |  |  |  | 1 |  |
| ST-53 complex | 53 | 1 | 0.9 |  |  |  |  | 1 | 1 |  |  |  |  |
| ST-167 complex | 1624 | 1 | 0.9 |  |  | 1 |  |  |  |  |  | 1 |  |
| ST-364 complex | 5771 | 1 | 0.9 | 1 |  |  |  |  |  | 1 |  |  |  |
| ST-865 complex | 4237 | 1 | 0.9 | 1 |  |  |  |  |  | 1 |  |  |  |
| ST-4821 complex | 11311 | 1 | 0.9 | 1 |  |  |  |  |  |  |  | 1 |  |
| No determine | 4953 | 2 | 1.7 |  | 2 |  |  |  |  | 1 |  | 1 |  |
|  | 276 | 1 | 0.9 | 1 |  |  |  |  |  |  |  |  | 1 |
|  | 1434 | 1 | 0.9 | 1 |  |  |  |  |  |  | 1 |  |  |
|  | 6610 | 1 | 0.9 |  | 1 |  |  |  |  |  |  |  | 1 |
| Total | | 115 | 100 | 78 | 29 | 6 | 1 | 1 | 25 | 29 | 19 | 37 | 5 |
