# Supplementary material for: Molecular characterization of Neisseria meningitidis isolates recovered from patients with invasive meningococcal disease in Colombia from 2013 to 2016
Source: PLoS One. 2020 Jul 14;15(7):e0234475. doi: 10.1371/journal.pone.0234475 (PMC7360035; doi:10.1371/journal.pone.0234475)
Supplement: Supplementary file 2 [file pone.0234475.s001.doc]

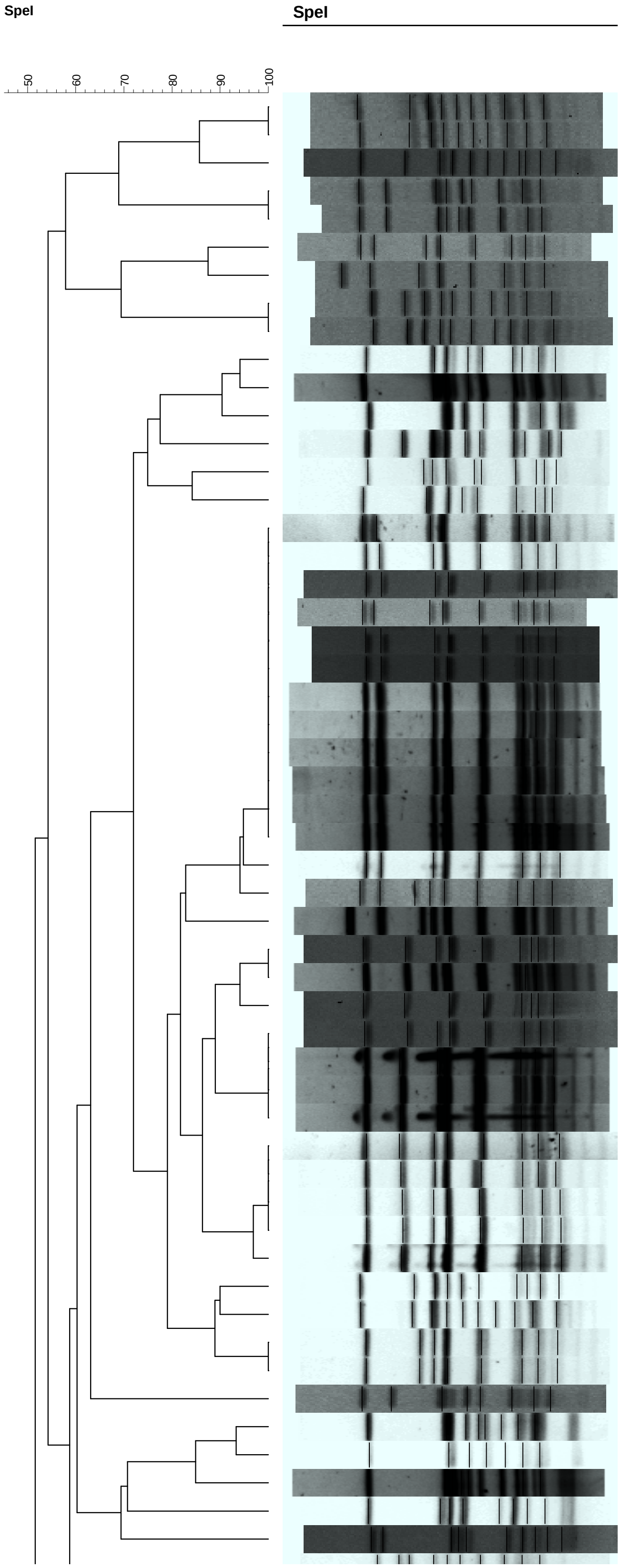

1046	C	2014	ANTIOQUIA	N	11149
999.2	B	2013	VALLE	N	485
1064	B	2015	BOGOTA	N	485
1087	B	2015	BOGOTA	.	9493
1143	B	2016	SUCRE	.	9493
1044	B	2014	BOLIVAR	.	9493
1124.2	B	2016	ANTIOQUIA	.	2561
1134.2	B	2016	BOGOTA	.	2288
1162	B	2016	VALLE	.	2288
1018	B	2014	BOLIVAR	M	9493
988	B	2013	BOGOTA	M	.
1016	B	2014	VALLE	M	5682
1027	B	2014	BOGOTA	.	3989/9493
1021	B	2014	ANTIOQUIA	.	9493
1029	B	2014	VALLE	.	409
1005	B	2014	ANTIOQUIA	B	9493
1015	B	2014	BOLIVAR	B	
1051.2	B	2015	BOLIVAR	B	9493
1069.3	B	2015	RISARALDA	B	9493
1076	B	2015	BOLIVAR	B	9493
1078	B	2015	BOLIVAR	B	9493
955	B	2013	BOLIVAR	B	
958	B	2013	BOLIVAR	B	
973	B	2013	BOLIVAR	B	
978	B	2013	BOLIVAR	B	9493
987	B	2013	BOLIVAR	B	
994	B	2013	BOLIVAR	B	9493
1032	B	2014	ANTIOQUIA	B	9493
1175	C	2016	BOGOTA	B	11
993	B	2013	BOLIVAR	B	9493
1066	B	2015	VALLE	B	9493
989	B	2013	BOLIVAR	B	9493
1065	B	2015	BOLIVAR	B	9493
1057	B	2015	BOLIVAR	B	9493
995	B	2013	ANTIOQUIA	B	9493
996	B	2013	ANTIOQUIA	B	9493
997	B	2013	ANTIOQUIA	B	9493
1006	B	2014	BOLIVAR	B	9493
1033	B	2014	BOLIVAR	B	9493
1035	B	2014	57	B	9493
1037	B	2014	57	B	9493
1036	B	2014	57	B	9493
1011	B	2014	SANTAND.	B	485
1012	B	2014	BOGOTA	B	485
1017	B	2014	BOLIVAR	B	9493
1019	B	2014	BOLIVAR	B	9493
1139.2					
1034	B	2014	RISARALDA	L	32
1038	B	2014	RISARALDA	L	32
981	B	2013	NTE/SANT.	L	33
1014	B	2014	SUCRE	.	33
1058	B	2015	BOLIVAR	.	33
[illegible]	[illegible]	[illegible]	[illegible]	[illegible]	[illegible]

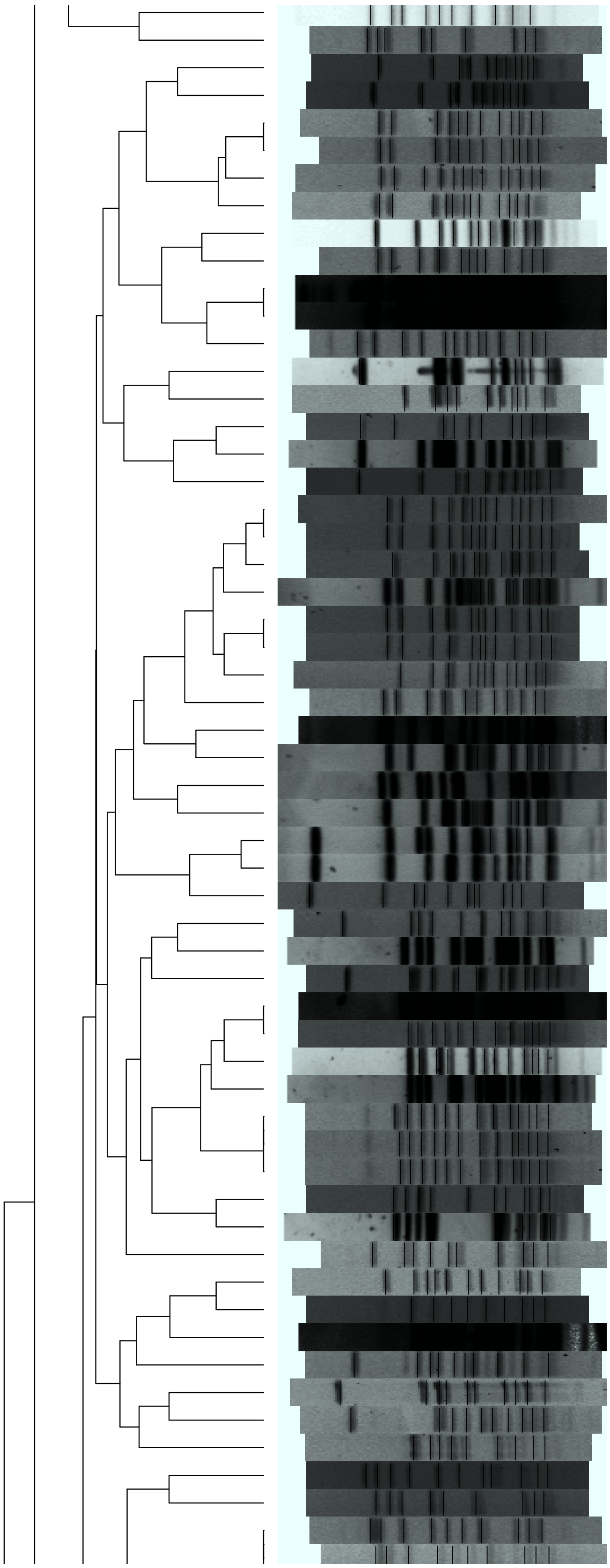

1020	B	2014	RISARALDA	.	4237
1137.2	Y	2016	ANTIOQUIA	.	1624
1121	C	2016	BOGOTÁ	NR	3128
1122	C	2016	BOGOTA	NR	2288
1173	Y	2016	VALLE	.	23
1041.3					
1068.2					
1045	C	2014	SANTAND.	.	
1028	B	2014	BOGOTA	.	4953/5771
1136.3					1478/2851
967	C	2013	NTE/SANT.	K	
983	C	2013	BOLIVAR	K	178
1159	C	2016	SANTAND.	K	
1004	B	2013	BOLIVAR	.	33
1040.2	B	2014	ANTIOQUIA	.	33
1088	C	2015	RISARALDA	O	11
992	B	2013	BOLIVAR	O	33
1114	B	2016	ANTIOQUIA	O	35
1061	Y	2015	ANTIOQUIA	D	
1106	Y	2016	BOGOTÁ	D	
1090	C	2015	ANTIOQUIA	D	
980	Y	2013	CAQUETA	D	
1107	C	2016	BOLIVAR	D	11
1109	C	2016	BOGOTÁ	D	
1086	Y	2015	BOGOTÁ	D	
1155	Y	2016	BOGOTA	D	23
1026	C	2014	ANTIOQUIA	.	
972	Y	2013	BOGOTA	.	
1031	W1.	2014	NO DATA	.	
990	Y	2013	BOGOTA	.	
1010	Y	2014	VALLE	J	
1025	Y	2014	RISARALDA	J	
1050.2	Y	2014	CAQUETA	J	5024
1085	C	2015	VALLE	NR	
977	B	2013	BOGOTA	.	1383
1091	B	2015	ANTIOQUIA	.	9493
1013	C	2014	BOGOTA	E	4953
1056	B	2015	ANTIOQUIA	E	3992
1003	B	2013	ANTIOQUIA	E	2288
984	B	2013	SANTAND.	E	3128
1055.3	C	2015	BOGOTA	E	
1166	C	2016	BOGOTA	E	
1168	C	2016	BOGOTA	E	4953
1097	C	2015	ANTIOQUIA	.	
971	B	2013	RISARALDA	.	9193
1151	B	2016	ANTIOQUIA	.	11311
1039	Y	2014	ANTIOQUIA 7	.	
1135	Y	2016	BOGOTA	.	.
1030	C	2014	NO DATA	.	
1171	B	2016	BOGOTA	.	32
1053	B	2015	BOLIVAR	.	7558/9493
1174	W	2016	ANTIOQUIA	.	184
986	NST	2013	RISARALDA	.	53
1079	C	2015	VALLE	.	
1140	C	2016	BOGOTA	NR	
1138.2	A	2016	BOGOTA	A	
1152	C	2016	SANTAND.	A	

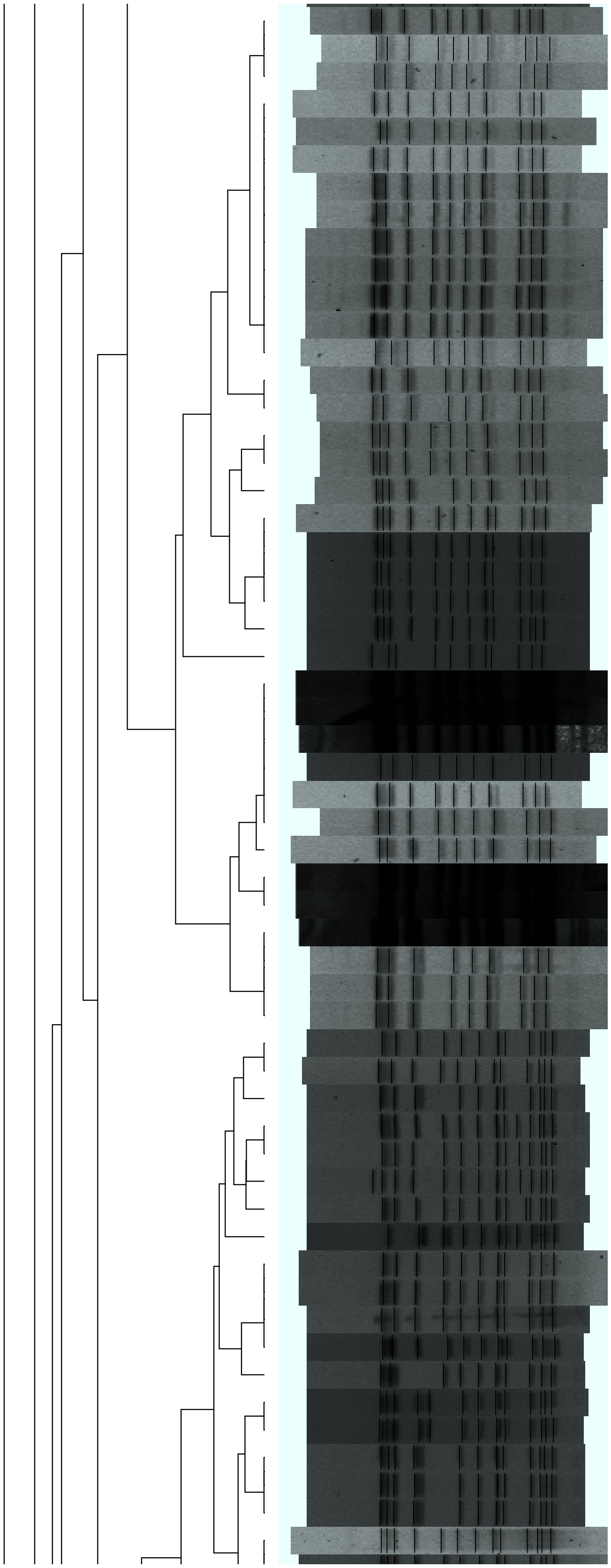

1138.2	A	2016	BOGOTA	A	
1152	C	2016	SANTAND.	A	
1150	C	2016	RISARALDA	A	11
1042	C	2014	META	A	
1081.2				A	.
1083.2	C	2015	QUINDIO	A	
1148	C	2016	RISARALDA	A	11
1149	C	2016	RISARALDA	A	11
1161	C	2016	BOGOTA	A	
1165	C	2016	BOGOTA	A	
1167	C	2016	BOGOTA	A	
1170	C	2016	CESAR	A	11
965	C	2013	ANTIOQUIA	A	
1141	A	2016	BOGOTA	A	
1145	C	2016	BOGOTA	A	
1127.3-1				A	.
1133.3-1				A	
1100.3-1				A	
1062.2				A	
1126	C	2016	BOGOTA	A	
1130	C	2016	BOGOTA	A	
1132	C	2016	VALLE	A	11
1125	C	2016	ANTIOQUIA	A	
1131	C	2016	BOGOTA	A	.
1000	C	2013	ANTIOQUIA	A	
1001	C	2013	ANTIOQUIA	A	
1024	C	2014	ANTIOQUIA	A	
1077	C	2015	ANTIOQUIA	A	11
1117.3	Y	2016	VALLE	A	11
1164.3				A	
1153.2				A	
1009	C	2014	ANTIOQUIA	A	
985	C	2013	ANTIOQUIA	A	
1023	C	2014	ANTIOQUIA	A	
1154	C	2016	CORDOBA	A	11
1157	C	2016	BOGOTA	A	
1158	C	2016	BOGOTA	A	
1094	C	2015	SANTAND.	C	
1110	C	2016	BOGOTÁ	C	.
1105	Y	2016	BOGOTÁ	C	23
1093	C	2015	META	C	
1095	C	2015	BOGOTÁ	C	11
1099	C	2015	BOGOTÁ	C	.
1096	C	2015	ANTIOQUIA	C	
1115	C	2016	VALLE	C	
1080	C	2015	META	C	
1082	C	2015	BOGOTÁ	C	.
1089	B	2015	ARAUCA	C	1434
1120	C	2016	BOGOTÁ	C	11
1098	C	2015	SANTAND.	C	
1112	C	2016	BOYACA	C	11
1113	C	2016	BOYACA	C	11
1101	C	2015	BOGOTÁ	C	.
1102	C	2015	BOGOTÁ	C	.
1103	C	2015	BOGOTÁ	C	11
1052	C	2015	ANTIOQUIA	C	
[illegible]	[illegible]	[illegible]	[illegible]	[illegible]	

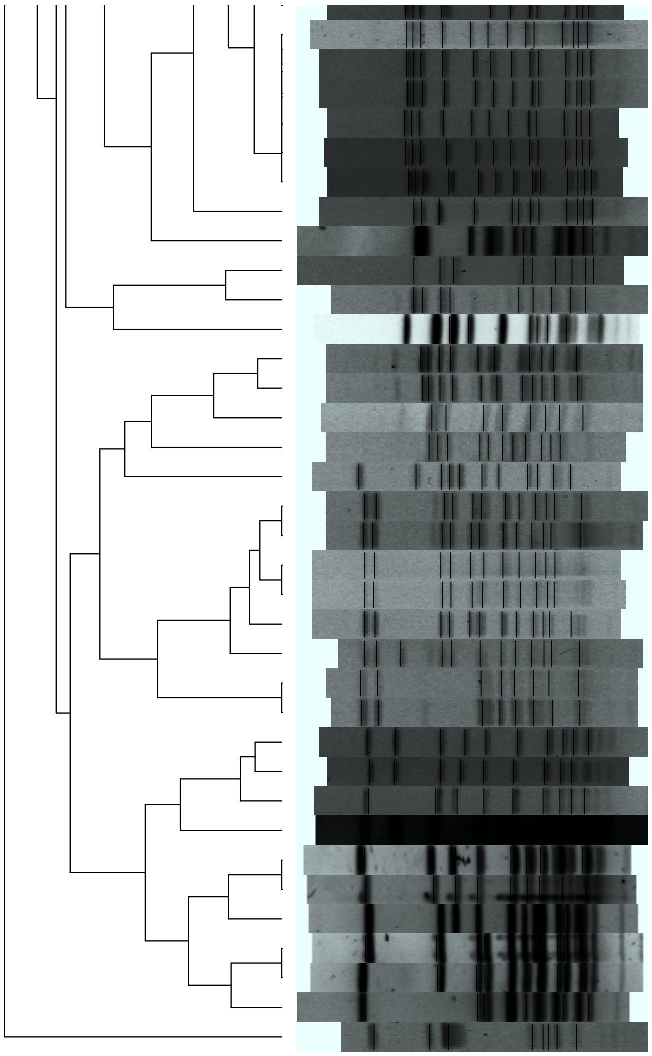

[illegible]	[illegible]	[illegible]	[illegible]	[illegible]	[illegible]
1052	C	2015	ANTIOQUIA	C	
1062	C	2015	ANTIOQUIA	C	
1063	C	2015	ANTIOQUIA	C	
1108	C	2016	BOGOTÁ	C	11
1111	C	2016	BOGOTÁ	C	.
1116	C	2016	BOYACA	C	11
1054.2	C	2015	NORTE DE.	C	
966	C	2013	ANTIOQUIA	.	
1049.2	B	2014	BOGOTA	.	213
1156	C	2016	SANTAND.	.	
1022	B	2014	ANTIOQUIA	.	
1169	B	2016	META	I	1383
1172	B	2016	META	I	1383
1177	B	2016	ANTIOQUIA	I	1383
979.2	C	2013	BOGOTA	.	3128
1043	B	2014	BOGOTA	.	9493
1160	C	2016	VALLE	G	
1163	C	2016	VALLE	G	2561
1060.3	C	2015	VALLE	G	
1067.3	C	2015	VALLE	G	
1092.2	C	2015	BOGOTA	G	
1144	C	2016	VALLE	G	2561
1119.2	B	2016	ANTIOQUIA	.	9461
1123	B	2016	ANTIOQUIA	.	9461
1059	C	2015	VALLE	H	278
1075	C	2015	VALLE	H	
1084	C	2015	VALLE	H	
1007	C	2014	BOGOTA	H	
969	B	2013	BOLIVAR	F	35
982	B	2013	BOLIVAR	F	35
991	B	2013	ATLANTICO	F	35
1002	B	2013	BOLIVAR	F	35
998	B	2013	MAGDALE.	F	35
10008/#1	B	2014	BOLIVAR	F	35
1047.3					.
